# Fractional order memcapacitive neuromorphic elements reproduce and predict neuronal function

**DOI:** 10.1038/s41598-024-55784-1

**Published:** 2024-03-09

**Authors:** Patricia Vazquez-Guerrero, Rohisha Tuladhar, Costas Psychalinos, Ahmed Elwakil, Maurice J. Chacron, Fidel Santamaria

**Affiliations:** 1 Department of Neuroscience, Developmental and Regenerative Biology, The University of Texas at San Antonio, San Antonio, TX 78349 USA; 2 Department of Physics, University of Patras, Patras, Greece; 3 Department of Electrical and Computer Engineering, University of Sharjah, PO Box 27272, Sharjah, UAE; 4 Department of Electrical and Software Engineering, University of Calgary, Calgary, AB T2N 1N4 Canada; 5 Department of Physiology, McGill University, Quebec, H3G 1Y6 Canada

**Keywords:** Neuroscience, Computational neuroscience, Dynamical systems, Electrical and electronic engineering

## Abstract

There is an increasing need to implement neuromorphic systems that are both energetically and computationally efficient. There is also great interest in using electric elements with memory, memelements, that can implement complex neuronal functions intrinsically. A feature not widely incorporated in neuromorphic systems is history-dependent action potential time adaptation which is widely seen in real cells. Previous theoretical work shows that power-law history dependent spike time adaptation, seen in several brain areas and species, can be modeled with fractional order differential equations. Here, we show that fractional order spiking neurons can be implemented using super-capacitors. The super-capacitors have fractional order derivative and memcapacitive properties. We implemented two circuits, a leaky integrate and fire and a Hodgkin–Huxley. Both circuits show power-law spiking time adaptation and optimal coding properties. The spiking dynamics reproduced previously published computer simulations. However, the fractional order Hodgkin–Huxley circuit showed novel dynamics consistent with criticality. We compared the responses of this circuit to recordings from neurons in the weakly-electric fish that have previously been shown to perform fractional order differentiation of their sensory input. The criticality seen in the circuit was confirmed in spontaneous recordings in the live fish. Furthermore, the circuit also predicted long-lasting stimulation that was also corroborated experimentally. Our work shows that fractional order memcapacitors provide intrinsic memory dependence that could allow implementation of computationally efficient neuromorphic devices. Memcapacitors are static elements that consume less energy than the most widely studied memristors, thus allowing the realization of energetically efficient neuromorphic devices.

## Introduction

Neuronal and artificial intelligence benefit from history-dependent self-adapting systems^1–16^. History-dependence explains neuronal dynamics from the movement of synaptic receptors to the generation of complex patterns of action potentials or spikes^2,4,17–22^. Our fractional order leaky integrate-and-fire (LIF) and Hodgkin–Huxley (HH) computer models reproduced a wide range of results of power-law history dependent firing rate and spike timing adaptation^21^. A fractional order differential equation is a non-local operator that involves intrinsic memory from previous activity^23^. Since power-law history-dependence in neurons show optimal coding properties^5,7^, it would be of importance to build neuromorphic circuits that implement such non-linear self-adapting mechanism. However, implementing a fractional order derivative in an electric circuit has been elusive, primarily due to the lack of electric elements with such properties^24^.

Electrical elements with memory, or memelements, are theoretic electric components whose intrinsic characteristics change with previous activity^25^. The most studied memelement for neuromorphic applications is the memristor, which has been realized with different materials^26–29^. A less studied element is the memcapacitor^30^. Memcapacitors could theoretically consume lower static power than memristors, see analysis in^31^ and other results^32–34^. Theoretical work shows that memelements can be described with fractional order integro-differential equations^35^. Within the physical foundations of the LIF and HH, the fractional order derivative corresponds to a fractional order capacitor. Thus, we propose that neuromorphic systems could be implemented using memcapacitors with fractional order differentiation properties.

In this work, we show that a particular electric element known as a super-capacitor^36^ has both, fractional differentiation and memcapacitive properties. Using super-capacitors we implemented a fractional LIF circuit^37^. The fractional LIF circuit replicates all the properties of our computer model that are also found in multiple brain areas^5,38–40^. We also implemented a fractional order HH circuit. This circuit showed distinct dynamics that were not previously described with our models, but that we were able to replicate. Under some input current conditions the circuit generated spiking dynamics consistent with a system in criticality. Both, the fractional LIF and HH circuits showed optimal coding properties. We found out that the critical dynamics of the fractional HH circuit reproduces the spontaneous and evoked activity of pyramidal neurons from the electro-sensory line lobe (ELL) in live immobilized weakly-electric fish (*Apteronotus leptorhynchus*). We used the fractional HH circuit to predict changes in spiking activity after prolonged stimulation, which we replicated experimentally. We also reproduced our results using a CMOS implementation of a fractional order capacitor^41^. Taken all together, our work shows that we can use fractional order memcapacitors to implement neuromorphic elements with intrinsic firing rate adaptation and optimal coding properties. This firing rate adaptation is the result of intrinsic memory and ***not*** a simple relaxation of the spiking mechanism. The realization of memcapacitive based neuromorphic elements could provide a platform for the implementation of systems that are energy and computationally efficient.

## Results

### A super-capacitor with fractional order differentiator and memcapacitive properties

The fractional order LIF model is^21^:

1 $$D^{\eta } v = G_{m} v + i \left( t \right)$$

where $v$ is voltage; ${G}_{m}$ is the membrane conductance; $i$ is current; $t$ is time; and ${D}^{\eta }$ is the Caputo definition of a fractional order derivative of order $\eta$:

2 $${D}^{\eta }v=\frac{1}{\Gamma \left(1-\eta \right)}{\int }_{0}^{t}\frac{{D}^{1}v\left(t\right)}{{\left(t-u\right)}^{\eta }}du$$

The ${\left(t-u\right)}^{-\eta }$ term is the intrinsic memory trace. The voltage resets at threshold (${v}_{Th}$) and has a refractory period (${t}_{r}$). The memory trace continuously integrates past activity.

The classic LIF is a model of current through a resistor and an ideal capacitor in parallel (Eq. 1 with $\eta =1)$^42^. The current, ${i}_{c}$, through the capacitor with capacitance ${C}_{o}$ is:

3 $${i}_{c}\left(t\right)={{C}_{o}D}^{1}v\left(t\right)$$

By analogy, in the fractional LIF model this corresponds to a fractional order capacitor:

4 $${i}_{c}\left(t\right)={C}_{o}{D}^{\eta }v\left(t\right)$$

Furthermore, real capacitors have an associated conductance (${G}_{c}$)^43^:

5 $${i}_{c}\left(t\right)={v}_{C}\left(t\right){G}_{c}+{C}_{o}{D}^{\eta }{v}_{c}\left(t\right)$$

Using a Laplace transform on both sides, re-arranging and applying an inverse Laplace transform the response of this circuit to a constant input current, ${i}_{c}\left(t\right)=I$ , is:

6 $${v}_{C}\left(t\right)={v}_{c}\left({t}_{0}\right)+I/{G}_{c}(1-{E}_{\eta }(-t/{\tau }_{C}))$$

With ${\tau }_{C}={C}_{o}/{G}_{c}$, and ${E}_{\eta }\left(z\right)$ the Mittag–Leffler function. At asymptotic times, Eq. 6 converges to a power-law:

7 $$v_{C} \left( t \right) = v_{C} \left( {t_{0} } \right) + I\left( {1/G_{c} + t^{\eta } /C_{o} \Gamma (1 + \eta )} \right)$$

When $\eta =1$ and ${{R}_{c}=1/G}_{c}=0$ the equation reduces to the ideal capacitor:

8 $$I={C}_{o}\frac{{v}_{C}\left(t\right)-{v}_{C}\left({t}_{0}\right)}{t}$$

**Figure 1:**
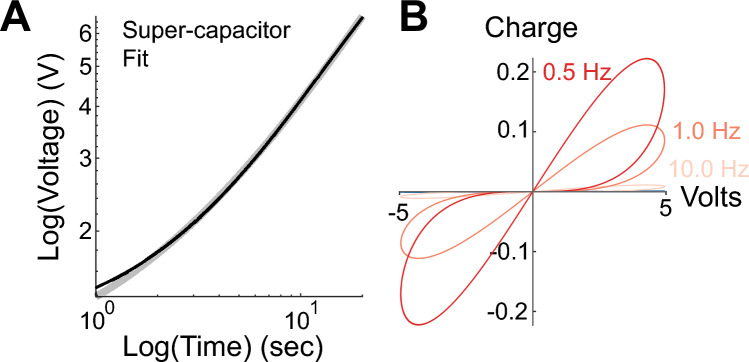
A super-capacitor has fractional order and memcapacitive properties. (**A**) Voltage versus Time Response to a $20 \text{ } \mu A$ step current for a super-capacitor stack (black) and fit to analytical solution (gray). (**B**) Voltage versus Charge plot in response to a voltage saw-tooth input at different frequencies. The stack consisted of four super-capacitors in series with an equivalent capacitance of 5.5 mF.

The charging and discharging properties of some super-capacitors follow fractional order dynamics^44^. Super-capacitors have capacitances orders of magnitude larger than traditional electrolytic capacitors because these elements are mainly designed for energy storage applications^45^. We connected in series four $22 \text{ }mF$ super-capacitors to reduce their capacitance ($5.5 \text{ }mF$ equivalent). Using Eq. 7 we determined that the super-capacitor stack had a fractional derivative order, $\eta = 0.82 \pm 2.20 \times 10^{ - 3} \;{ }95\%\;{\text{CI}}$ (Fig. 1A)^43^.

A memcapacitor is defined as^25,30^:

9 $$q=C\left(x,v,t\right)v \text{ and }{D}^{1}x=f(x,v,t)$$

where $q$ is electric charge; $C$ is capacitance with units of $F/x\cdot t$; and $x$ is an internal variable (such as the flux, $\varphi$). From the definition of flux:

10 $${D}^{1}\varphi =v$$

If we assume that $C\left(\varphi ,v,t\right)={C}_{0}\varphi$, then

11 $${\text{q}}={C}_{0}{\int }_{0}^{t}vd\tau \cdot v$$

Equation 11 results in a hysteresis curve pinched at the origin. Indeed, the measured $q$ vs.$V$ curve of the super-capacitors (Fig. 1B) shows hysteresis pinched at the origin and a frequency dependent amplitude, both properties of memcapacitors^25^. Thus, the super-capacitors have fractional order derivative and memcapacitive properties.

### A fractional order leaky integrate-and-fire analog circuit

**Figure 2:**
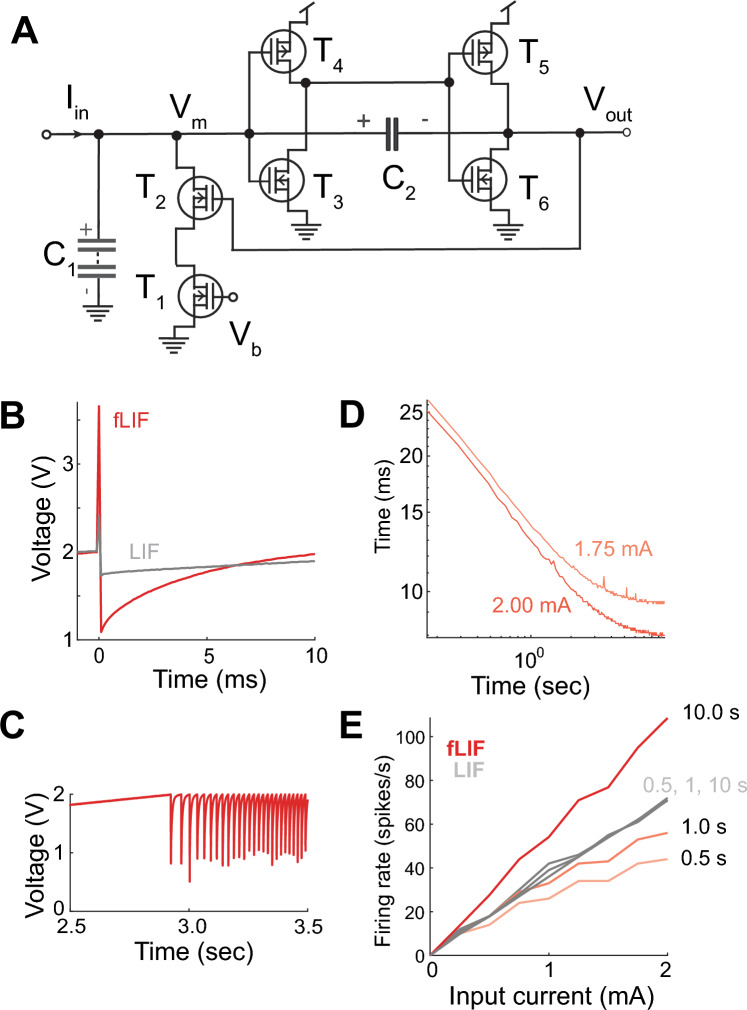
The fractional leaky integrate-and-fire circuit. (**A**) Schematic diagram. (**B**) Action potential generated by the classical (LIF) and fractional LIF (fLIF) circuits. (**C**) Example of spike train generated in response to a $2 \text{ }mA$ constant input. Spikes were clipped for clarity. (**D**) Inter-spike interval as a function of time and constant input current. (**E**) Firing rate in the LIF and fLIF circuits. The firing rate in the fLIF circuit was calculated using different time windows from the onset of the first spike.

We implemented an analog circuit of a LIF neuron^37^ (Fig. 2A). The circuit generates spikes of less than $0.1 \text{ } ms$ with ${v}_{th}=2 \text{ }V$. We substituted ${C}_{1}=0.1 \text{ } \mu F$ with the super-capacitor stack. When using an electrolytic $5 \text{ } mF$ capacitor the LIF circuit failed to generate action potentials ($0.1-10 \text{ }mA$ input), not shown. However, the circuit with the super-capacitor stack generated spikes with the same ${v}_{th}$ but with higher amplitude (Fig. 2B). This could be because the charging of super-capacitors is much slower than electrolytic capacitors thus allowing the circuit to function.

The classic LIF generates action potentials at a constant rate when stimulated with constant current (not shown). By contrast, the fractional LIF shows a delay in generating the first spike, followed by a power-law decrease in the inter-spike intervals (ISIs, Fig. 2C,D). The combination of slowed onset in firing and acceleration of the ISI affects the calculation of the firing rate from the time of onset of the first spike (Fig. 2E)^21^. However, at longer stimulation times, the firing rate of the fractional LIF circuit becomes faster than for the classical case. We have described this process with our computer model in which the intrinsic memory trace acts as a feedback mechanism that feeds into increasing firing rates^21^.

**Figure 3:**
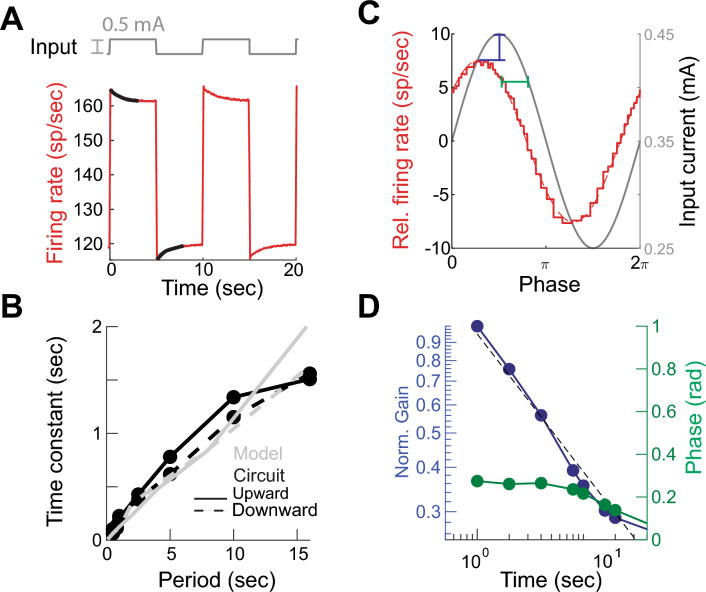
History-dependence and fractional differentiation by the fractional leaky integrate-and-fire (fLIF) circuit. (**A**) Firing rate in response to square-wave input. (**B**) Fitted time constants to the firing rate accommodation to square inputs in (**A**) for different periods. There is a fit for the upward and downward adaptations—black traces in (**A**). (**C**) Example of firing rate in response to oscillatory input of 4 s period. (**D**) Firing rate gain and phase shift with respect to sinusoidal inputs of different periods.

We wanted to test how previous activity affected the firing rate adaptation of the fractional LIF circuit^5^. We injected square-wave inputs of different frequencies into the fractional LIF circuit (Fig. 3A). The DC and amplitude of input signal were adjusted to always generate action potentials. We calculated the time constant of instantaneous firing rate adaptation for the positive and negative parts of the cycle as a function of period length by fitting a single exponential to each phase of the cycle (Fig. 3A black). This resulted in a time constant of adaptation that depended on the previous period length, a non-linear phenomenon observed in real neurons^5,38^ (Fig. 3B black). The fractional LIF model reproduced this behavior ($\eta =0.2$, Fig. 3B gray). Thus, the firing rate of the fractional LIF shows history dependence.

We wanted to determine if the fractional order properties of the super-capacitors were reflected in the firing rate activity of the neuron. If the firing rate, $f$, of a neuron encodes the input, ${I}_{n}={\text{Asin}}(2\pi \omega t),$ as a fractional order derivative, then^46^:

12 $$f=\frac{{d}^{\eta }{I}_{n}}{d{t}^{\eta }}\to f={\text{A}}{\left(2\mathrm{\pi \omega }\right)}^{\eta }{\text{sin}}\left(2\pi \omega t+\frac{\eta \pi }{2}\right)$$

We injected a sinusoidal input to the fractional LIF neuronal circuit. The amplitude and DC components were set to generate spikes at all input values. We varied the input wavelength ($\lambda =1/\omega$) from $0.1 \text{ }s$ to $40 \text{ }s$ and fitted a sine function to the instantaneous firing rate (Fig. 3C). This shows that the firing rate amplitude decays as a power-law, ${\eta }_{G}= 0.39\pm 0.07 \text{ } 95 \% \text{ } CI$, and the phase shift is fixed ${\eta }_{\phi }= 0.32\pm 0.04 \text{ }SEM$, Fig. 3D. Thus, the spiking of the fractional LIF circuit has fractional order derivative properties.

**Figure 4:**
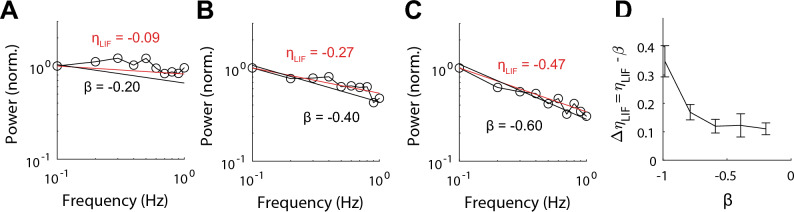
Power spectrum whitening by the fractional leaky integrate-and-fire (LIF) circuit. (**A**–**C**) Power spectrum of the firing rate (o), with power-law exponent of the fit (${\eta }_{LIF}$) and the power-spectrum of the pink-noise input signal (solid black) characterized by its exponent,$\beta$. (**D**) Value of ${\Delta \eta }_{LIF}={\eta }_{LIF}-\beta$ as a function of $\beta$. Error bars correspond to SEM.

The power-spectrum of multiple natural signals have a power-law structure ($1/{f}^{\beta }$) also known as pink-noise^7,47–56^. From Eq. 12 if $\beta =\eta$ and ${\text{A}}{=(2\mathrm{\pi \omega })}^{-\beta }$ then the power spectrum of the firing rate should be flat, ${\eta }_{LIF}\sim 0$, also known as ***spectrum whitening*** which is a tale-tale of optimal coding computations^57,58^. The same additive properties predict a constant shift in the exponent of the firing rate power spectrum, ${\Delta \eta }_{LIF}={\eta }_{LIF}-\beta$. We calculated ${\Delta \eta }_{LIF}$ to inputs with pink-noise (range 0.01 to 1.00 Hz) and varying $\beta$ values. (Fig. 4A–D). This analysis shows that for $\beta =[-0.2, -0.4, -0.6]$ the value ${\Delta \eta }_{LIF}$ is constant. For values of $\beta <-0.6$ the system becomes less sensitive to the input. The same analysis shows that the firing rate spectrum is flat when $\beta =-0.2$ (${\eta }_{LIF}=-0.09 \pm 0.14 \text{ } 95 \% \text{ } CI$).

### The fractional differentiation properties of the leaky integrate-and-fire circuit depend on the fractional order of the super-capacitors

**Figure 5:**
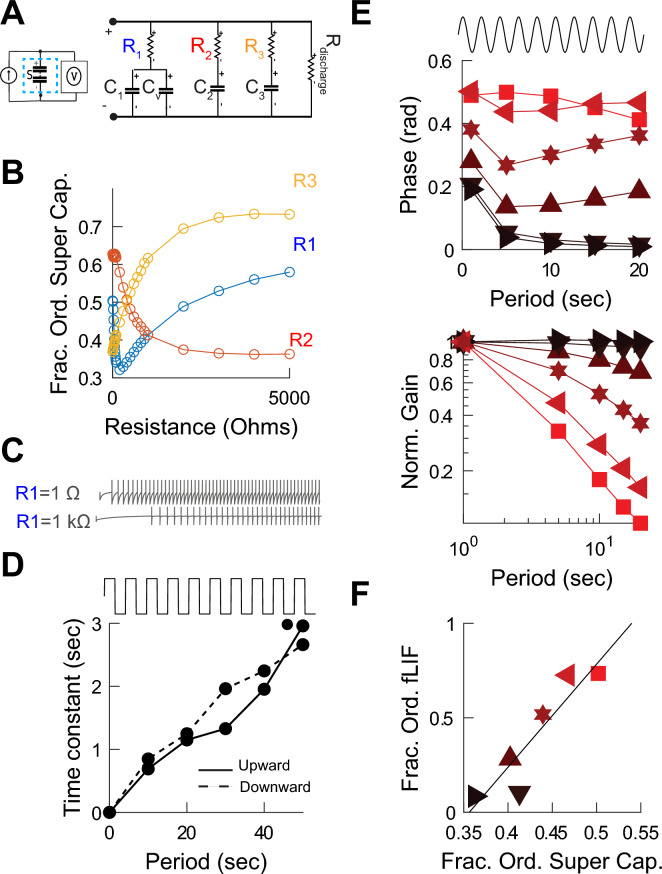
The fractional Leaky Integrate-and-Fire (fLIF) analog circuit model. (**A**) Circuit model of a super-capacitor. (**B**) Calculated super-capacitor fractional order as a function of internal variables shown in (**A**). (**C**) Examples of spiking responses of the fLIF circuit model to constant input. (**D**) Time constant of adaptation of the modeled fLIF in response to square-wave input of different periods. (**E**) The firing rate phase and gain in response to sinusoidal current input of different periods and as a function of the order of the fractional order of the super-capacitor. (**F**) Relationship between the fractional orders of the spiking response calculated from (**E**) and the fractional order of the super-capacitor.

We used an analogue circuit simulation platform to study the effect of fractional oder super-capacitors of different orders on the responses of the fractional LIF circuit (see Methods). To do this we used an available super-capacitor model^59,60^ (Fig. 5A). We systematically varied the values of the resistances in the model and characterized the resulting fractional order (Fig. 5B). We then built an analog circuit simulation of the fractional LIF. The model showed spike latencies (Fig. 5C), firing rate adaptation to square inputs (Fig. 5D), and fractional derivative properties (Fig. 5E) as a function of the fractional order of the super-capacitor (Fig. 5F).

### A fractional order Hodgkin–Huxley circuit shows criticality and optimal coding

#### Criticality in the fractional order Hodgkin–Huxley circuit

**Figure 6:**
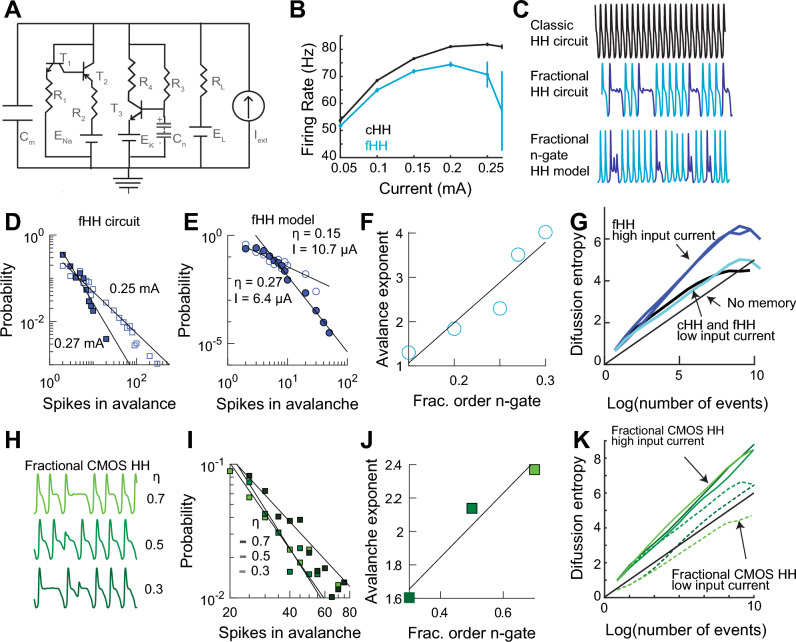
The fractional order Hodgkin and Huxley (fHH) circuit. (**A**) Circuit diagram, C_n_ is a stack of super-capacitors. (**B**) Firing rate vs input current for the classical (cHH) and fHH circuits. (**C**) Examples of spike trains of the cHH and fHH circuits (0.27 mA in both), and the fractional n-gate HH computer model (10.7 µA and η = 0.15). (**D**) Histogram of avalanches in the fHH circuit for two input currents. (**E**) Histograms generated by the fractional order computer model. (**F**) Avalanche exponent vs. value of η of the n-gate in the fHH computer model. (**G**) Diffusion entropy analysis of the fHH circuit under different input current states. (**H**) Spike trains when using a CMOS implementation of a fractional order capacitor with the HH circuit, $I = 0.3 \text{ }mA$. (**I**) Avalanche histograms generated by the CMOS fHH circuit. (**J**) Avalanche exponent as a function of the fractional order of the CMOS capacitor from (**I**). (**K**) Diffusion entropy analysis of the CMOS fHH circuit.

Voltage activated membrane conductances show history-dependence^61^. To study history-dependence in a biophysical model of neuronal activity we implemented a circuit of the classic HH model^62^ (Fig. 6A). The circuit generated spikes that closely matched the HH model (Sup. Mat. Fig. S1). Following on our previous studies of a fractional HH computer model^20^ we added to the $n$-gate part of the circuit a stack of five super-capacitors ($47 \text{ }mF$ each) in series ($9.40 \text{ }mF$ equivalent) with fractional order of ${\eta }_{2} = 0.93 \pm 0.01 \text{ }95\% \text{ } CI$ (Sup. Mat. Fig. S2). The fractional and classic circuits had the same ${v}_{th}$. At high currents ($>0.25 \text{ }mA$) the fractional HH circuit showed increased variability and reduction in firing rate (Fig. 6B). This was because the circuit generated a mixture of normal and *complex spikes*, spikes containing a plateau potential of varying periods (Fig. 6C) that lasted for as long as the stimulus was on (not shown).

The fractional HH computer model replicated the complex spikes (Sup. Mat. Fig. S3) using $\eta =0.2-0.3$ for the $n$-gate. A phase plane plot of the measured and modeled complex spikes shows mixed mode oscillations with a smaller attractor representing the plateau (Sup. Mat. Fig. S4)^20^ known as a canard^63,64^. Thus, the complex spikes emerge from the internal dynamics of the system and not from some saturation in the circuit.

We hypothesized that the apparent random nature of the complex spikes was because the circuit was at criticality^65^. We defined an avalanche as the number of simple spikes between two complex spikes. The histogram of avalanches follows a power-law, consistent with a system in criticality (Fig. 6D). This was also replicated in the model with the fractional order of the $n$-gate linearly related to the avalanche structure (Fig. 6E,F). Thus, complex spikes emerge when the system is at criticality.

Diffusion entropy analysis characterizes stochastic processes with memory^66–68^. This analysis shows that the classical and fractional HH at low-input current have no memory. By contrast, the circuit at criticality shows history-dependence (Fig. 6G). The difference in dynamics in the fractional HH circuit is probably due that at low input current the dynamics are controlled by the electrolytic capacitor and at higher input current (and voltages) the super-capacitors dominate the dynamics.

Instead of super-capacitors we used a CMOS implementation of fractional order capacitors^41^, see Supplementary Material Fig. S5. For the three different values of fractional order implemented in the chip the HH circuit generated avalanches and the spiking activity showed diffusion entropy history-dependence (Fig. 6H–K).

Fractional order capacitance is also found in skin, fruits, and vegetables, due to their fractal cellular structures^69–71^. To generalize our results, we implemented the circuit using dried fruit, $\eta =0.67\pm 0.03 \text{ } 95\% \text{ }CI$. Under such arrangement the HH circuit generated spikes and power-law ISIs (Sup. Mat. Fig. S6). Thus, collectively, our results show that fractional order capacitance, independent of its physical nature, is sufficient to generate fractional order spiking activity.

#### Transition from simple to complex spiking suggests a change in coding

**Figure 7:**
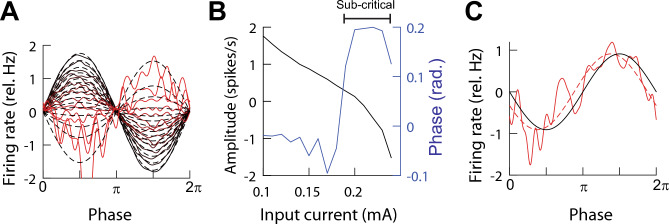
The sub-critical state of the fractional Hodgkin–Huxley (fHH) circuit. (**A**) Relative firing rate (solid) in response to $1 \text{ }Hz$ frequency and $0.01 \text{ }mA$ amplitude input signal with varying DC values ($0.10,\text{ black}-0.25\text{ mA},\text{ red}$). Dashed lines are fits. (**B**) The amplitude (gain) and phase shift with respect to input signal from (**A**). (**C**) Phase advance in the sub-critical regime $(DC = 0.26 \text{ }mA$ and $0.05 \text{ }mA$ amplitude at $1 \text{ }Hz$). The phase advance results in a fractional order derivative of $\eta =0.23\pm 0.01 \text{ } 95\% \text{ CI}$. Firing rate, solid red; fit, dashed red; input, solid black.

We characterized the firing rate of the circuit to sinusoidal input with fixed amplitude ($AC = 0.05 \text{ }mA$) and frequency ($1 \text{ }Hz$) and varying DC (Fig. 7A). The amplitude of the firing rate decreases and flips signs as DC values increase while the phase switches from negative to positive (Fig. 7B,C). The flipping of the firing rate amplitude is because of widening of the spikes without becoming complex spikes ($<0.22 \text{ }mA$). In the presence of complex spikes, there was no phase lag (not shown). We called the regime where there is positive phase in the absence of complex spikes the ***sub-critical*** phase. The reversal in amplitude can be interpreted as a change in coding the input from the firing rate to the ISI (Eq. 12).

### The fractional Hodgkin–Huxley circuit reproduces and predicts sensory processing and critical dynamics of neurons from the live weakly-electric fish

#### Fractional differentiation, optimal coding, and criticality in fish and circuit

**Figure 8:**
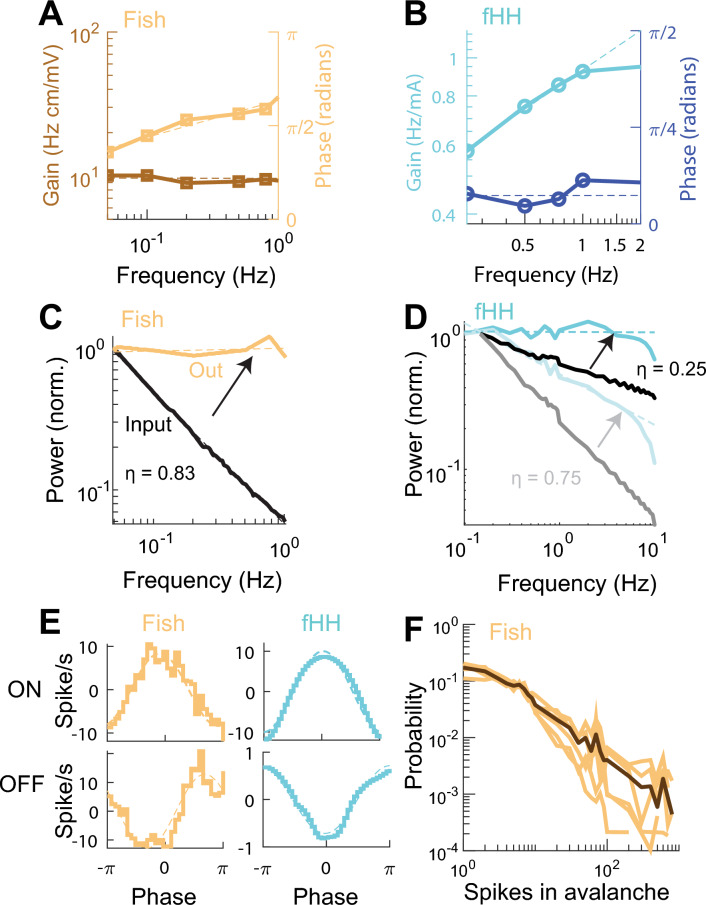
The fractional Hodgkin–Huxley (fHH) circuit reproduces optimal coding properties of the weakly-electric fish neurons. (**A**) Firing rate gain and phase shift of ELL neurons in the weakly electric fish in response to sinusoidal stimulation. (**B**) Gain and phase advance in the fHH circuit in the sub-critical state. (**C**) Power-spectrum of firing rate of a LS neuron (brown) in response to pink-noise of order $\eta = 0.83$ electric field stimulation (black). (**D**) Power spectrum of firing rate (blue) of the fHH circuit in response to pink-noise of order 0.25 and 0.75. (**E**) ELL neurons and fHH circuit showing ON and OFF responses to sinusoidal input. (**F**) Histograms of avalanches recorded from in vivo electric fish at rest (brown) and their average (black).

Neurons in weakly-electric fish perform a fractional differentiation of modulated electrical stimulation with a gain characterized by ${\eta }_{ELLg}=0.26\pm 0.09 \text{ } 95\% \text{ } CI$, and phase advance of ${\eta }_{ELL\phi }=0.43\pm 0.02 \text{ }SEM$ (Fig. 8A) mediated by a potassium conductance^48^. We replicated this behavior in the fractional HH circuit in the sub-critical state with an input current with DC of $2.4 \text{ }\mu A$ and amplitude of $0.05 \text{ }mA$ over a range of 0.25 to 2.00 $Hz$ (Fig. 8B). The power-law gain, ${\eta }_{HHg}=0.33\pm 0.08 \text{ } 95\% \text{ }CI$, and the phase advance ${\eta }_{HH\phi }=0.26 \pm 0.03 \text{ }SEM$ had values very close to those measured experimentally.

The same neurons that perform a fractional order differentiation show spectrum whitening of pink-noise signals. When some LS neurons from the ELL are stimulated with pink-noise with ${\beta }_{In}=-0.83$ the power-spectrum of the firing rate is flat, ${\eta }_{ELLout}=0.02 \pm 4x{10}^{-3} \text{ }95\%\text{ } CI$ , Fig. 8C ^7^. We stimulated the fractional HH circuit with pink-noise with $\beta =[-0.2, -2]$. It was when we used a pink-noise with $\beta =-0.2$ that the exponent of the firing rate was flat, ${\eta }_{HHout}= -0.07 \pm 0.21 \text{ } 95\% \text{ }CI$. We also calculated the power-spectrum to pink-noise with $\beta =-0.75$ expecting a power-spectrum ${\eta }_{HHout}=-0.55$, the experimental results were close to this value, ${\eta }_{HHOut}=-0.41\pm 0.07 \text{ } 95\% \text{ }CI$ (Fig. 8D).

Without changing any parameters in the fractional HH circuit, we tested if we could reproduce other properties of ELL neurons. For example, neurons that show fractional order differentiation can be classified as ON or OFF cells. The ON cells follow the input, while OFF cells decrease their firing when the input is high. Our fractional HH circuit at low input current ($DC = 1.0 \text{ }mA$, amplitude $0.05 \text{ }mA$) reproduces the ON behavior and without fractional order differentiation. In the sub-critical regime ($DC = 2.4 \text{ }mA$, amplitude $0.05 \text{ }mA$), the circuit shows fractional differentiation and OFF behavior (Fig. 8E).

At rest, the neurons of the ELL generate spiking bursts^72^. We hypothesized that the burst were equivalent to complex spikes observed in the circuit. Indeed, the avalanches, number of spikes between bursts, in the weakly-electric fish have a power-law distribution with an exponent of $-0.92\pm 0.06 \text{ } 95\% \text{ }CI$ (Fig. 8F).

#### The fractional HH circuit predicts long-term spike time adaptation

**Figure 9:**
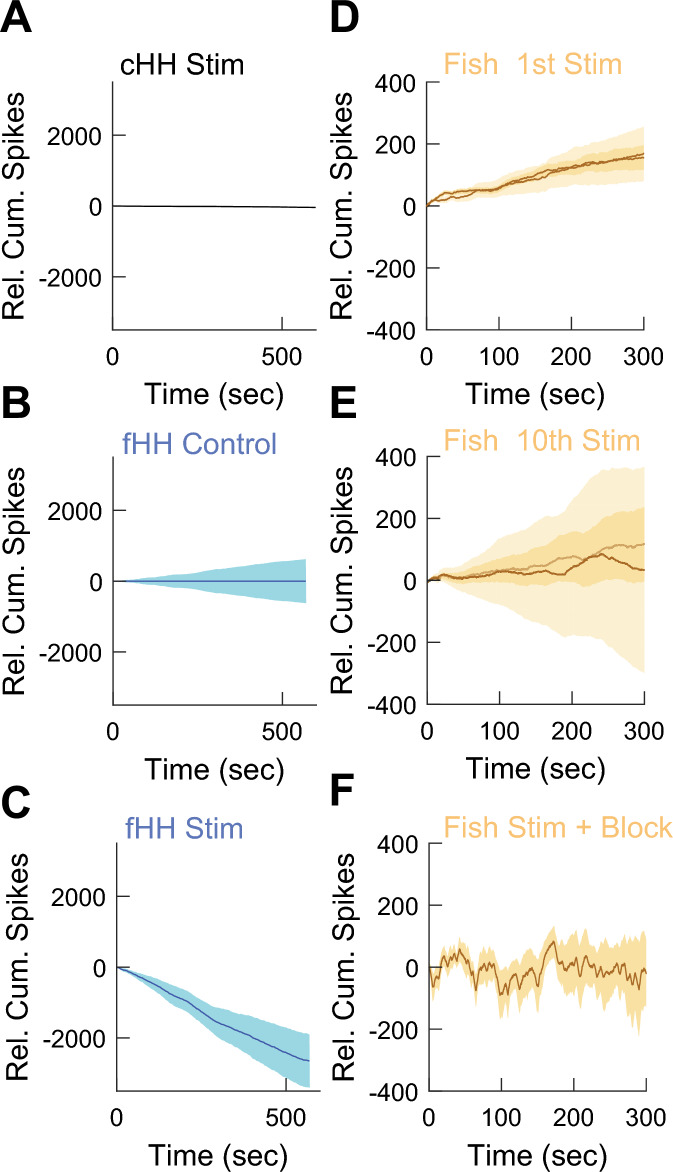
Long term history-dependence in the fractional HH (fHH) circuit in criticality and in the weakly electric fish ELL neurons. All panels: relative cumulative spikes with respect to control conditions after delivering a stimulus or control. (**A**) Classic HH circuit (cHH) after stimulus. (**B**) In the absence of a stimulus (control) in the fHH circuit in criticality. Shaded areas correspond to 95% CI. (**C**) As in B but after delivering a 30 s stimulus. (**D**) Average cumulative relative spikes from two different experiments in weakly electric fish after delivering a 30 s stimulus. (**E**) Responses of the same neurons in D but after the 10th repetition of the stimulus and recording epoch. (**F**) Average cumulative responses from a fish after blocking feedback signals to the ELL.

History-dependence should result in inputs affecting the spiking of the neuron for periods of time longer than its characteristic time constants. Using the circuit in normal, sub-critical, or critical regimes we delivered a stimulus (lower the input current for 30 s but high enough to keep firing) and monitored the spiking for 10 min. We calculated the relative cumulative spikes between the post- and pre-stimulus times (see Methods). The classical (Fig. 9A) and fractional sub-critical (not shown) circuits showed no changes in spiking activity (horizontal lines). By contrast, when we performed the measurements with the stimulus the circuit showed a prolonged and robust decrease in firing (Fig. 9B,C).

While recording from the live fish, we delivered a masking electric stimulus that inhibit ELL cells, similar to the ‘low’ input current in the HH circuit. We calculated the cumulative spikes in control and stimulus conditions. This shows that ELL neurons respond to the masking stimulus with a robust increase in the cumulative spikes (Fig. 9D). The neurons continue to respond to subsequent stimulus over multiple repetitions. After the 8th period, the neurons show no change in cumulative spiking with respect to control (Fig. 9E).

Past work shows that fractional order optimal coding properties of ELL pyramidal cells are strongly influenced by both feedback input from nucleus praeminentialis (nP) as well as serotonergic input^73^. We bilaterally blocked feedback signals and repeated the experiment showing no change in cumulative firing (Fig. 9F). Taken together, our results show that primary sensory neurons show long lasting intrinsic memory that is consistent with their fractional order properties.

## Discussion

In this work, we showed that an off-the-shelf super-capacitor is a memcapacitor and a fractional order differentiator. We then used our theoretical framework of fractional order neurodynamics^20,21,74^ to implement fractional order LIF and HH circuits. These circuits replicate non-linear properties of single neurons observed in multiple brain areas and animal species. Of particular importance is that fractional order dynamics provides a natural mechanism for optimal coding of signals characterized by power-laws, which are common in natural and constructed environments^53,75–81^. Remarkably, we did not tune any of the parameters of our circuits to any particular experimental results. Thus, showing that fractional order differentiation could be a robust neuromorphic implementation that encompasses the history-dependence properties of real neurons.

### Super-capacitors as fractional order memelements and a platform for efficient neuromorphic systems

Super-capacitors are distinct from electrolytic capacitors due to their large energy density^82^. The large majority of super-capacitors achieve high capacitances due to the use of porous materials like activated carbon obtained from natural sources^82^. The super-capacitors employed in this work use this technology. These super-capacitors have an electric double-layer between a solid electrode and an aqueous electrolyte (for a review refer to^83^). A parameter of great importance for super-capacitors is the minimum voltage necessary to activate the electrolytic reaction, known as the cell voltage. Our super-capacitors have a cell voltage of $1.7 \text{ }V$^84^. This is the voltage at which fractional order differentiation and then the appearance of complex spikes is observed in the fractional HH circuit (Fig. 7B, transforming the DC applied voltage). Thus, the emergence of history dependent properties of the circuit is explained by the activation of the super-capacitors.

The super-capacitors had a fixed fractional order intrinsic to their physical properties. While this is a current challenge in fabrication, there are promising new technologies^41,44,85–88^, including their miniaturization^86^. In any case, we analyzed the dependence of the fractional differentiation of the firing rate with respect to the fractional order of the super-capacitor using circuit models or CMOS implementations. In both cases, we linked the properties of the memcapacitor to the coding properties of the neuron. Our research supports efforts to develop fractional order super-capacitors with varying orders and miniaturized to allow the implementation of neuromorphic devices^31^.

We demonstrated that the fractional order properties of the circuit are not exclusive to electric implementations. Instead, by using a CMOS circuit, we showed that any fractional order dielectric could provide the dynamics necessary to implement fractional order neuromorphic systems. We consider our results using dried fruit as a further demonstration that any dielectric with fractional order properties could provide functionality to neuromorphic devices; however, we are well aware that these measurements have low precision and uniformity due to the nature of the natural product. In any case, our work supports research in the development of biomimetic memcapacitors^89,90^.

Memcapacitors can consume lower static power than memristors, and thus have the potential for building neuromorphic systems orders of magnitude more energy-efficient^31–34^. This is because capacitors are electric field based, instead of current based. Initial work with neural networks not only supports this idea, but also suggests that they could be computationally efficient^91–93^. Thus, fractional-order memcapacitros could be a platform for energetically and computational efficient neuromorphic systems.

### Further development of fractional order LIF and HH systems

The fractional order LIF reproduced all the properties of our previously published computer model^21^. However, there were some limitations. The fractional differentiation required that the input current had a relatively small amplitude, suggesting that some other parts of the circuit dominate its dynamics at larger input amplitudes. It was not our objective to maximize the effect of any part of the circuit, but to demonstrate the possibility of implementing fractional neurodynamics in neuromorphic circuits. In any case, our work using the circuit model analysis provides the building blocks to optimize parameters for particular input ranges and tasks.

The fractional HH circuit has three states: normal, sub-critical, and critical. Our previous computational work suggested the emergence of complex patterns and complex spikes as a function of input current and time^20^. However, computational limitations did not allow us to investigate our computer model for periods comparable to what we reported here. Thus, showing another advantage of a neuromorphic approach.

The classical regime is consistent with low input current that does not engage the super-capacitor properties. This requires further study in relation to super-capacitor physical properties and design. Another area of study is the sub-criticality regime where we showed fractional order differentiation. In this regime we interpreted that the signal was being encoded by the ISI instead of the firing rate. This requires experimental corroboration and an understanding of how neuromorphic systems could operate in this regime. Finally, at higher input currents, the circuit generated simple and complex spikes. The complex spikes were reproduced by our models and are consistent with the emergence of a canard. This dynamics requires further study in the context of chaotic systems^94–96^ and the physical and intrinsic memory properties of the super-capacitors^44^. In all cases, it is necessary to determine the energy consumption of the circuits and compare to other implementations.

The rich dynamics shown by the fractional HH circuit reminded us of the spiking properties of ELL neurons in the weakly-electric fish. These neurons show fractional differentiation and optimal coding properties in the live animal receiving naturalistic stimulation. The fractional HH circuit was not capable to reproduce all properties of all the neurons in only one of its states. However, we were able to match the behavior of different real neurons with the circuit in the classical, sub-critical, or critical state. While the matches were not identical, the overall properties are captured by our neuromorphic element. The important point is that the response to the dynamical input depends on the bias constant current in which this is mounted, opening the possibility of using the same neuron to perform different computational tasks dynamically.

Instead of using the circuits to replicate published work, we used the fraction HH circuit to predict the behavior of ELL neurons in the live fish. Our results show that the spiking activity of these neurons retain the effect of a stimulus for multiple minutes. Furthermore, our experimental results show that this adaptation is over tens of minutes and reflects adaptation at much larger scales that previously thought. These results challenge current models of primary sensory neurons^97,98^. Traditionally, these neurons are expected to transmit input reliably and fast to the rest of the nervous system. The fact that they show long-term adaptation to previous input calls into question current ideas of coding in which these neurons are assumed, essentially, memoryless.

Recent directions in neuromorphic spiking circuit is to reduce the use of capacitor or fully eliminate them^99^. However, systems made on memcapacitive technology could be at least 8 × more energetically efficient that with resistive devices^31^ while other theoretical work suggest that memcapacitive reservoir computing could be efficient computationally efficient^91^. Future work should address the trade-offs between the computational properties of fractional order capacitors against their impact on area efficiency in circuit design and operation speed of complex neuromorphic hardware.

### Perspective

A grand challenge of Artificial Intelligence and engineering is to build systems that are concurrently energetically and computationally efficient^100^. Our work further supports the use of memcapacitors by focusing on those elements that also implement a fractional order differentiation. By combining memcapacitive and fractional differentiation properties these systems could be energy efficient and computationally optimal for the types of signals commonly found in nature. Our results also show that neuromophic elements that reproduce fractional order neurodynamics can be used to predict the responses of real neuronal systems, thus providing a promising platform for brain-machine interfaces^101^.

## Methods

### Super-capacitors, circuits, and computer models

#### Super-capacitors

For all of our circuits we used NEC/TOKIN (TOKIN, Japan) super-capacitors. For the LIF circuit we used 22 mF super-capacitors (part # FS0H223ZF) rated at 5.5 V and 60 Ω maximum equivalent series resistance (ESR) at 1 kHz. Similarly, for the HH circuit we used 47 mF super-capacitors (part # FYH0H473ZF) also rated at 5.5 V but with 100 Ω maximum ESR at 1 kHz. Both types of super-capacitors show fractional order dynamics^44^.

#### The fractional leaky integrate-and-fire model and circuit

The fractional order LIF is^21^ (Eq. 5):

13 $${D}^{\eta }{v}_{m}=-({v}_{m}-{v}_{r})/{{\tau }_{m}}^{\eta }+I/{c}_{m}$$

where ${v}_{m}$ is the membrane voltage, ${v}_{r}$ is the resting voltage, ${\tau }_{m}= {r}_{m}{c}_{m}$ is the membrane time constant, with ${r}_{m}$ the membrane resistance and ${c}_{m}$ the membrane conductance. The conductance is ${{g}_{m}=1/r}_{m}$. The input current is $I$. The neuron generates a spike when ${v}_{m}\ge {v}_{Th}$ and the voltage is clamped to ${v}_{r}$ for a refractory period, ${t}_{r}$. The fractional order of the derivative is $0<\eta \le 1$.

We used the Grunwald–Letnikov integration of the fractional derivative

14 $${v}_{m}\left({t}_{N+1}\right)={\left(\Delta t\right)}^{\eta }\frac{\left(-\left({v}_{m}\left({t}_{N}\right)-{v}_{r}\right)+{r}_{m}I\left({t}_{N}\right)\right)}{{\tau }_{m}}+\sum_{k=0}^{N}{{c}_{k}^{\eta }v}_{m}\left({t}_{N-k}\right)$$

With the integration time $\Delta t=0.1 \text{ }ms$. The memory weights are

15 $${c}_{k}^{\eta }=\left(1-\frac{1+\eta }{k}\right){c}_{k-1}^{\eta }$$

With ${c}_{0}^{\eta }=\eta$. The system can also be solved using the L1 scheme (see below). We determined that a memory window longer than 10 s did not change the results.

The original LIF circuit is the well-known design by Carver Mead (Fig. 2A). The circuit uses MOSFET transistors. Transistors ${T}_{1}$, ${T}_{2}$, ${T}_{3}$ and ${T}_{6}$ were p-channel; and ${T}_{4}$ and ${T}_{5}$ were n-channel. The ${T}_{1}$ and ${T}_{2}$ transistors implement a resetting of the voltage, V_m_, that is due to charge accumulation of capacitor C_1_. The transistor circuits ${T}_{3} -{T}_{4}$ and ${T}_{5} -{T}_{6}$ implement current mirrors that generate the spike. The original circuit had the following characteristics ${C}_{1}=0.1 \text{ }\mu F$; $C2 = 22 \text{ }\mu F$; $Vb = 5 \text{ }V$. The source of ${T}_{4}$ and ${T}_{5}$ were at $5 \text{ }V$. The spiking traces analyzed were measured from $Vm$. As described in the results, ${C}_{1}$ was substituted with a stack of super-capacitors in series.

#### Hodgkin–Huxley model and circuit

The Hodgkin–Huxley model is:

16 $${c}_{m}{D}^{1}{v}_{m}=-\left({g}_{m}\left({v}_{m}-{v}_{r}\right)+\overline{{g }_{K}}{n}^{4}({v}_{m}-{E}_{K})+\overline{{g }_{Na}}{m}^{3}h({v}_{m}-{E}_{Na})\right)+I$$

where $\overline{{g }_{K}}$ is the total potassium conductance; $\overline{{g }_{Na}}$ is the total sodium conductance;$n$

is the activation potassium gate; $m$ is the sodium activation gate; and $h$ is the sodium inactivation gate. The general form of the gating variables is

17 $${D}^{1}x={\alpha }_{x}\left(V\right)\left(1-x\right)-{\beta }_{x}\left(V\right)x$$

where $x = [ n, m, h ]$, the function ${\alpha }_{x}$ is the forward rate, and ${\beta }_{x}$ is the backward rate. The functional forms of $n$, $m$, and $h$ are

18 $$\begin{aligned} & \alpha_{n} \left( V \right) = \frac{{0.1 - 0.01\left( {V - V_{0} } \right)}}{{e^{{1 - 0.1\left( {V + V_{0} } \right))}} - 1}} \\ & \beta_{n} \left( V \right) = 0.125e^{{ - \frac{{V - V_{0} }}{80}}} \\ & \alpha_{m} \left( V \right) = \frac{{2.5 - 0.1\left( {V - V_{0} } \right)}}{{e^{{2.5 - 0.1\left( {V - V_{0} } \right))}} - 1}} \\ & \beta_{m} \left( V \right) = 4e^{{ - \left( {V - V_{0} } \right)/18}} \\ & \alpha_{h} \left( V \right) = 0.07e^{{ - \frac{{V - V_{0} }}{20}}} \\ & \beta_{h} \left( V \right) = \frac{1}{{1 + e^{{\left( {3 - 0.1\left( {V - V_{0} } \right)} \right))}} }} \\ \end{aligned}$$

The fractional order gating dynamics is

19 $${D}^{\eta }x={\alpha }_{x}\left(V\right)\left(1-x\right)-{\beta }_{x}(V)x$$

In this work we concentrated in studying the effects of fractional order in the potassium n-gate.

The normal parameter values were ${c}_{m}= 0.47 \text{ }\mu F$, $\overline{{g }_{Na}}= 120\text{ } mS$, $\overline{{g }_{k}}= 36\text{ } mS$, $\overline{{g }_{m}}= 0.3\text{ } mS$, ${E}_{Na}= 50 \text{ }mV$, ${E}_{K}= -77 \text{ }mV$, and ${v}_{r} = -65 \text{ }mV$. However, to replicate the criticality behavior (Fig. 6E) we modified the model with the following parameters: ${c}_{m}= 1 \text{ }\mu F$,$\overline{{g }_{Na}}= 70\text{ } mS$, $\overline{{g }_{k}}= 5\text{ } mS$, and ${E}_{K}= -77 \text{ }mV$, ${v}_{r} = -54.4 \text{ }mV$.

For these simulations we used a Runge–Kutta level 4 for the integer derivative equations and an L1 scheme for the fractional order potassium gate:

20 $$n\left({t}_{N+1}\right)={\left(\Delta t\right)}^{\eta }\Gamma \left(2-\eta \right)\frac{\left({n}_{\infty }\left({v}_{m}\left({t}_{N}\right)\right)-n\left({t}_{N}\right)\right)}{{\tau }_{n}\left({v}_{m}\left({t}_{N}\right)\right)}-\sum_{k=0}^{N}\left({\left(N+1-k\right)}^{1-\eta }-{\left(N-k\right)}^{1-\eta }\right)\left(n\left({t}_{N}\right)-n\left({t}_{N-1}\right)\right)$$

with

21 $${n}_{\infty }=\frac{{\alpha }_{n}}{{\alpha }_{n}+{\beta }_{n}} {; \tau }_{n}=\frac{1}{{\alpha }_{n}+{\beta }_{n}}$$

We used a previously published design of a circuit that can replicate the Hodgkin–Huxley model^62^, Fig. 6A. This circuit has four branches where each part of the circuit is equivalent to a part of the model. The two branches in the middle correspond to the sodium and potassium conductances. The original circuit has the following parameter values ${C}_{m}= 0.1 \text{ }\mu F$, ${r}_{l}= 100 \text{ }k\Omega$, ${E}_{l}= 0 \text{ }V$,${E}_{Na}= 5 \text{ }V$, ${R}_{1}= 100 \text{ }\Omega$, ${R}_{2}= 100 \text{ }\Omega$, ${R}_{3}= 1 \text{ }k\Omega$, ${R}_{4}= 674 \text{ }\Omega$, ${E}_{K}= 1.1\text{ } V$. The capacitor that controlled the potassium conductance had an original value of ${C}_{n} = 0.1 \text{ }mF$. For the fractional HH circuit, we connected Cn in series with five super-capacitors.

#### Measurements

We controlled the delivery of current or voltage and data acquisition with a National Instruments card (DAQ, PCI-6071E). We provided the current to our circuit with the DAQ connected to an Analog Stimulus Isolator (A-M Systems, model 2200). The membrane potential was acquired with an analog input of the same DAC, and the information was processed using LabVIEW (National Instruments, TX).

### Weakly-electric fish experiments

#### Animals

All animal procedures were approved by McGill University’s animal care committee and were performed in accordance with the guidelines of the Canadian Council on Animal Care. The study is reported in accordance with ARRIVE guidelines (https://arriveguidelines.org). Specimens of either sex of the wave-type weakly electric fish *Apteronotus leptorhynchus* (N = 7) were used exclusively in this study. Animals were purchased from tropical fish suppliers and were housed in groups (2–10 individuals) at controlled water temperatures (26–29 °C) and conductivities (100–800 µS*cm^−1^) according to published guidelines^102^. It was not possible to determine the age of the specimens used.

#### Surgery

Surgical procedures have been described in detail previously^103,104^. Briefly, 0.1–0.5 mg of tubocurarine (Sigma) was injected intramuscularly to immobilize the animals for electrophysiology experiments. The animals were then transferred to an experimental tank (30 cm x 30 cm x 10 cm) containing water from the animal’s home tank and respired by a constant flow of oxygenated water through their mouth at a flow rate of 10 mL*min^−1^. Subsequently, the animal’s head was locally anesthetized with lidocaine ointment (5%; AstraZeneca), the skull was partly exposed, and a small window (~ 5 mm^2^) was opened over the hindbrain as well as bilaterally over both midbrains in order to access nucleus praeeminentialis (nP) for drug application as described elsewhere^103,104^.

#### Stimulation

The electric organ discharge of *Apteronotus leptorhynchus* is neurogenic, and therefore is not affected by injection of curare. All stimuli consisted of amplitude modulations (AMs) of the animal’s own EOD and were produced by triggering a function generator to emit one cycle of a sine wave for each zero crossing of the EOD as done previously^105^. The frequency of the emitted sine wave was set slightly higher (~ 30 Hz) than that of the EOD, which allowed the output of the function generator to be synchronized to the animal’s discharge. The emitted sine wave was subsequently multiplied with the desired AM waveform (MT3 multiplier; Tucker Davis Technologies), and the resulting signal was isolated from the ground (A395 linear stimulus isolator; World Precision Instruments). The isolated signal was then delivered through a pair of chloritized silver wire electrodes placed 15 cm away from the animal on either side of the recording tank perpendicular to the fish’s rostro-caudal axis. This stimulation protocol has been used by multiple previous studies (see, e.g.,^106,107^). We used stimuli consisting of a 5–15 Hz noise (4th order Butterworth) carrier waveform (i.e., AM) whose amplitude (i.e., envelope) was further modulated sinusoidally at 0.05, 0.1, 0.25, 0.5, 1, 2 Hz to mimic the envelope signals due to relative movement between two fish^108–110^. Alternatively, we delivered a step increase in EOD amplitude lasting 30 s and repeated 20 times at intervals of 300 s. The data in Fig. 8 was extracted from the plots in their corresponding publication^7^.

#### Recordings

The data presented in Fig. 8A,D,G were performed with simultaneous extracellular recordings from ELL pyramidal cells within the lateral segment using metal-filled micropipettes as described previously^7,103–105,111,112^. We used CED 1401-plus hardware and Spike II software (Cambridge Electronic Design, Cambridge, UK) to record the resulting signal with resolution 0.1 ms.

For the experiments in Figs. 8F and 9 simultaneous extracellular recordings from ELL pyramidal cells were made using Neuropixels probes (Imec Inc.) as done previously^113–116^. The probe was angled at approximately 15 deg. with respect to vertical and advanced between 1500 and 2000 µm into the ELL with reference to the probe tip. Neurons were recorded at depths between 200 and 1400 µm from the brain surface and as such most likely included neurons within the lateral and centrolateral segments^117^. Recordings were digitized at 30 kHz using spikeGLX (Janelia Research Campus) and stored on a hard drive for further analysis. Spikes were sorted using Kilosort^118^ and subsequently curated using the phy application (https://github.com/cortex-lab/phy) ^118,119^.

#### Pharmacological inactivation

To study the effects of descending pathways, we recorded ELL pyramidal cells and behavior for control in conjunction with their responses after bilateral lidocaine (1 mM) injection into nP. Drug application pipettes were made using single-barrel borosilicate capillary glass tubes (OD 1.5 mm, ID 0.86 mm, A-M Systems) and pulled by a vertical micropipette puller (Stoelting) to a fine tip that was subsequently broken to attain a tip diameter of approximately 5 μm. All pharmacological injections were performed approximately 1250–1750 μm below the surface where the nP is located using a duration of 130 ms at ~ 20 psi using a Picospritzer (General Valve) as done previously^103,104,120^. It is important to note that the effects of such inactivation start within at most 30 s after injection and last throughout the experiment.

### Code and analysis

We developed custom made MATLAB (Natick, MA) code to simulate the spiking models, and to analyze all the simulations and experiments. We stimulated and acquired the data from the circuit with a National Instruments data acquisition card (DAQ, PCI-6071E). We provided the current to our circuit with the DAQ connected to an Analog Stimulus Isolator (A-M Systems, model 2200). The membrane potential was acquired with an analog input of the same DAC, and the information was processed using LabVIEW. We report 95% confidence intervals (95% CI) and Standard Error of the Mean (SEM).

#### Diffusion entropy analysis

Diffusion Entropy Analysis (DEA) was originally introduced by Allegrini et al.^66^. Modified versions of DEA have been applied recently^67,68^. We consider a diffusion process given by

22 $$\begin{array}{*{20}c} {x\left( t \right) = \mathop \smallint \limits_{0}^{t} \xi \left( {t^{\prime } } \right)dt^{\prime } } \\ \end{array}$$

This trajectory is converted into many diffusional trajectories using a moving window ‘*l*’ to record $\Delta x\left( t \right) = x\left( {t + l} \right) - x\left( t \right)$. For different values of t, it is equivalent to generating many Gibbs copies from which we can get the probability distribution function (PDF) p(x,l). We can then evaluate the Shannon entropy of the process using,

23 $$\begin{array}{*{20}c} {S\left( l \right) = - \mathop \smallint \limits_{ - \infty }^{\infty } p\left( {x,l} \right)\ln p\left( {x,l} \right)dl} \\ \end{array}$$

If the PDF is a Gaussian function, then $\delta = 0.5.$ If it is a Lévy function, it fits the scaling property,

24 $$\begin{array}{*{20}c} {p\left( {x,l} \right) = \frac{1}{{l^{\delta } }}F\left( {\frac{x}{{l^{\delta } }}} \right) } \\ \end{array}$$

where $\delta$ is the anomalous scaling $\delta > 0.5.$ Using Eq. 24 and 25 in the asymptotic limit $l \to \infty$ we get,

25 $$\begin{array}{*{20}c} {S\left( l \right) = A + \delta lnl} \\ \end{array}$$

where A is a constant. Note that when the PDF departs from the ordinary Gaussian case, it has slow tails with an inverse power-law function.

## Supplementary Information

Supplementary Information.

## Data Availability

All data collected and data analyses are available in GitHub (Github.com/SantamariaLab) or by request to Dr. Fidel Santamaria (fidel.santamaria@utsa.edu).
