# Author Correction: Fractional order memcapacitive neuromorphic elements reproduce and predict neuronal function

**DOI:** 10.1038/s41598-024-76011-x

**Published:** 2024-10-15

**Authors:** Patricia Vazquez‑Guerrero, Rohisha Tuladhar, Costas Psychalinos, Ahmed Elwakil, Maurice J. Chacron, Fidel Santamaria

**Affiliations:** 1 Department of Neuroscience, Developmental and Regenerative Biology, The University of Texas at San Antonio, San Antonio, TX 78349 USA; 2 Department of Physics, University of Patras, Patras, Greece; 3 Department of Electrical and Computer Engineering, University of Sharjah, PO Box 27272, Sharjah, UAE; 4 Department of Electrical and Software Engineering, University of Calgary, Calgary, AB T2N 1N4 Canada; 5 Department of Physiology, McGill University, Quebec, H3G 1Y6 Canada

Correction to: *Scientific Reports* 10.1038/s41598-024-55784-1, published online 09 March 2024

The Funding section in the original version of this Article was incomplete.

“National Institute of Health grant 1R01EB026939 and NSF-EFRI 2318139 to FS. CONACyT (Mexico) scholarship 625406 to PVG. Canadian Institutes of Health Research to MJC.”

now reads:

“National Institute of Health grant 1R01EB026939, NSF-EFRI 2318139, and NSF-2332744 to FS. CONACyT (Mexico) scholarship 625406 to PVG. Canadian Institutes of Health Research to MJC.”

The original Article has been corrected.

