# Supplementary material for: Fractional order memcapacitive neuromorphic elements reproduce and predict neuronal function
Source: Sci Rep. 2024 Mar 9;14:5817. doi: 10.1038/s41598-024-55784-1 (PMC10925066; doi:10.1038/s41598-024-55784-1)
Supplement: Supplementary file 1 — Supplementary Information. [file 41598_2024_55784_MOESM1_ESM.pdf]

Supplementary materials

Fractional order memcapacitive neuromorphic elements reproduce and predict neuronal function

Patricia Vazquez-Guerrero[1], Rohisha Tuladhar[1], Costas Psychalinos[2], Ahmed Elwakil[3,4], Maurice J. Chacron[5], and Fidel Santamaria[1*]
1 Department of Neuroscience, Developmental and Regenerative Biology. The University of Texas at San Antonio, San Antonio, Texas 78349.
2 Department of Physics, University of Patras, Greece.
3 Department of Electrical and Computer Engineering, University of Sharjah, PO Box 27272, Sharjah, UAE
4 Department of Electrical and Software Engineering, University of Calgary, T2N 1N4, Alberta, Canada
5 Department of Physiology, McGill University, Quebec, Canada H3G 1Y6
* Corresponding author: fidel.santamaria@utsa.edu

Action potentials generated by the classical Hodgkin-Huxley circuit and computer model

We implemented a circuit that replicated the Hodgkin-Huxley model. We also implemented the computer model (see Methods) and compared the spike shapes generated by both under constant current stimulation (Fig. S1).

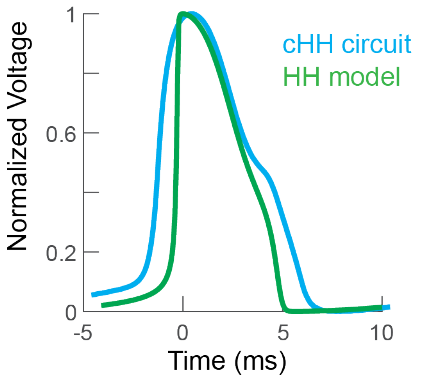

Figure S1. Spikes generated by the circuit (cyan) and computer model (green) of the Hodgkin-Huxley system of equations.

Fractional order of super-capacitors used in the Hodgkin-Huxley circuit

Following the same measurements performed in the main text we measured the fractional derivative order of a super-capacitor stack (Fig. S2).

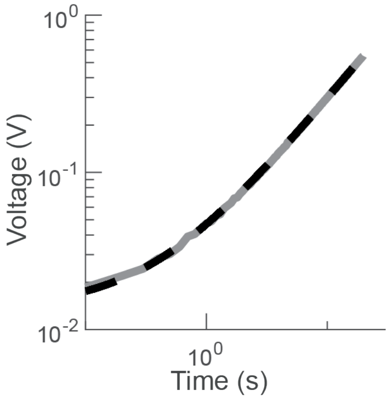

Figure S2. Voltage across a stack of super-capacitors after a current step stimulus. Measurement in black, fit from analytical solution in dashed gray.

The complex spikes in the fractional order Hodgkin-Huxley circuit and model

In what we describe as the critical state of the fractional Hodgkin-Huxley circuit the system generates complex spikes. The complex spikes were replicated with the computer model when considering fractional order dynamics in the n-gate (Fig. S3). The phase plane analysis shows two distinct oscillatory patterns that are consistent with a canard (Fig. S4). See main text for details.

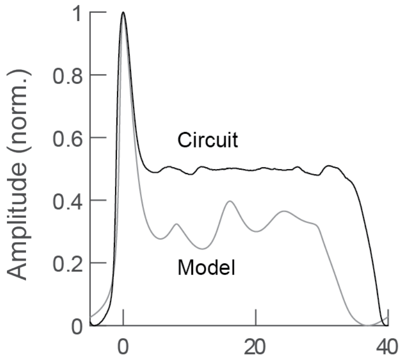

Figure S3. Complex spikes generated by the fractional order Hodgkin-Huxley circuit and computer model. The fractional order was implemented in the n-gate in both cases.

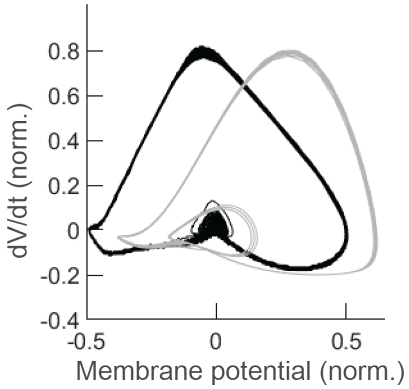

Figure S4. Phase plane analysis of the complex spikes generated by the Hodgkin-Huxley circuit (black) and compute model (gray.

The CMOS circuit

We used a CMOS implementation of the mathematical description of a fractional order capacitor. A photograph of the circuit and general description are in Figure S5. A detailed explanation has been previously published(1).

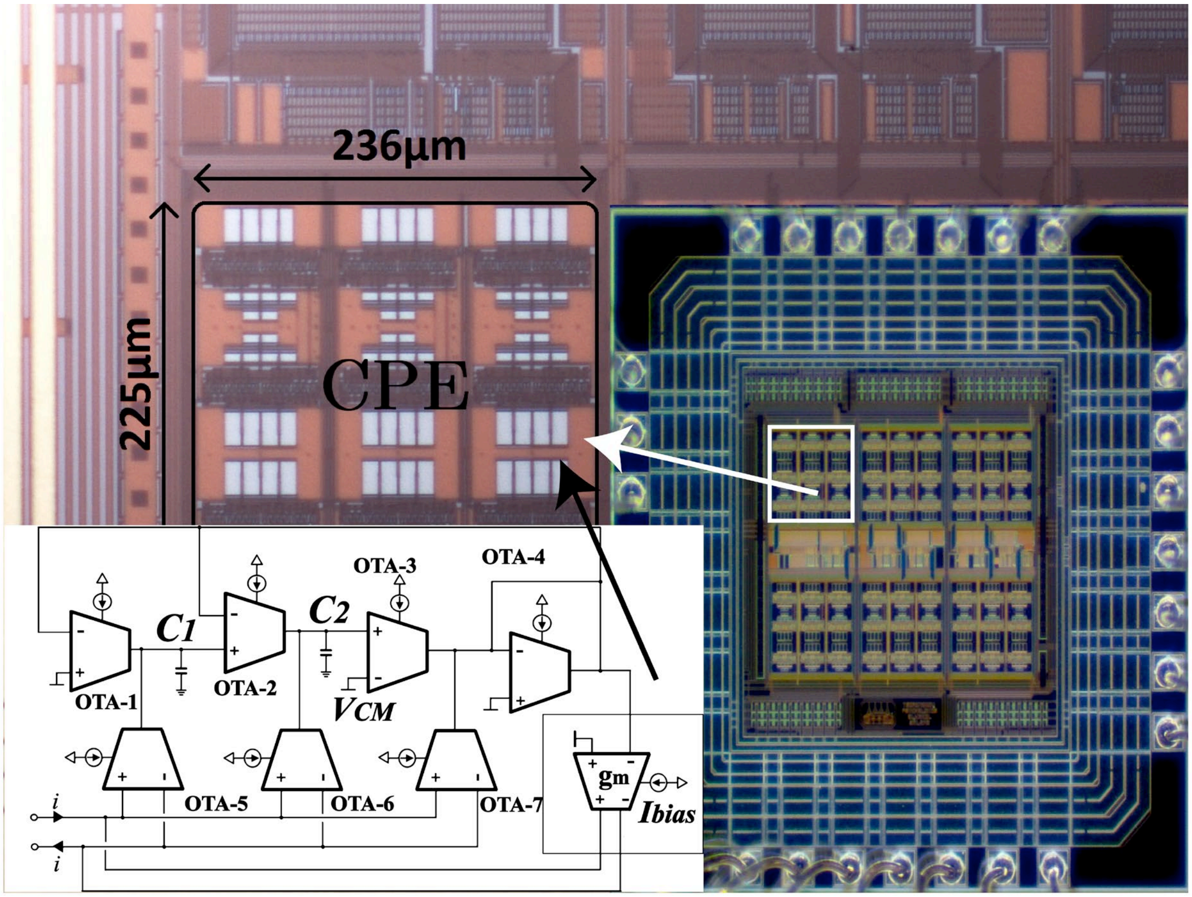

Figure S5. The fractional order CMOS circuit.

Implementing the fractional Hodgkin-Huxley circuit using other fractional order materials

It is widely known that fruits, plants, and vegetables have fractional order capacitance(2-7). We implemented the fractional Hodgkin-Huxley (HH) circuit using a pitted prune (Sunsweet, CA). The fractional order of the pitted prune was 0.67 ± 0.03 95% CI. We connected the prune to the circuit by inserting electrodes on the prune. For one configuration (Fig S6A) we needed to inject current in the hundreds of micro-Amperes to generate spikes (Fig. S6B). Depending on the input current the inter-spike interval (ISI) varied randomly (Fig. S6C). In contrast with the super-capacitor circuit the firing rate decreased as a function of input current with an increase in the variability (Fig. S6E). We repeated the measurements in a second configuration with electrodes farther apart (Fig. S6F). In this configuration we used less than 8 μA to generate action potential that resembled complex spikes (Fig. S6G). As in the first configuration, the ISI appeared random (Fig. S6H) and had a long tail distribution (Fig. S6I). The firing rate decayed as a function of input current (Fig. S6J).

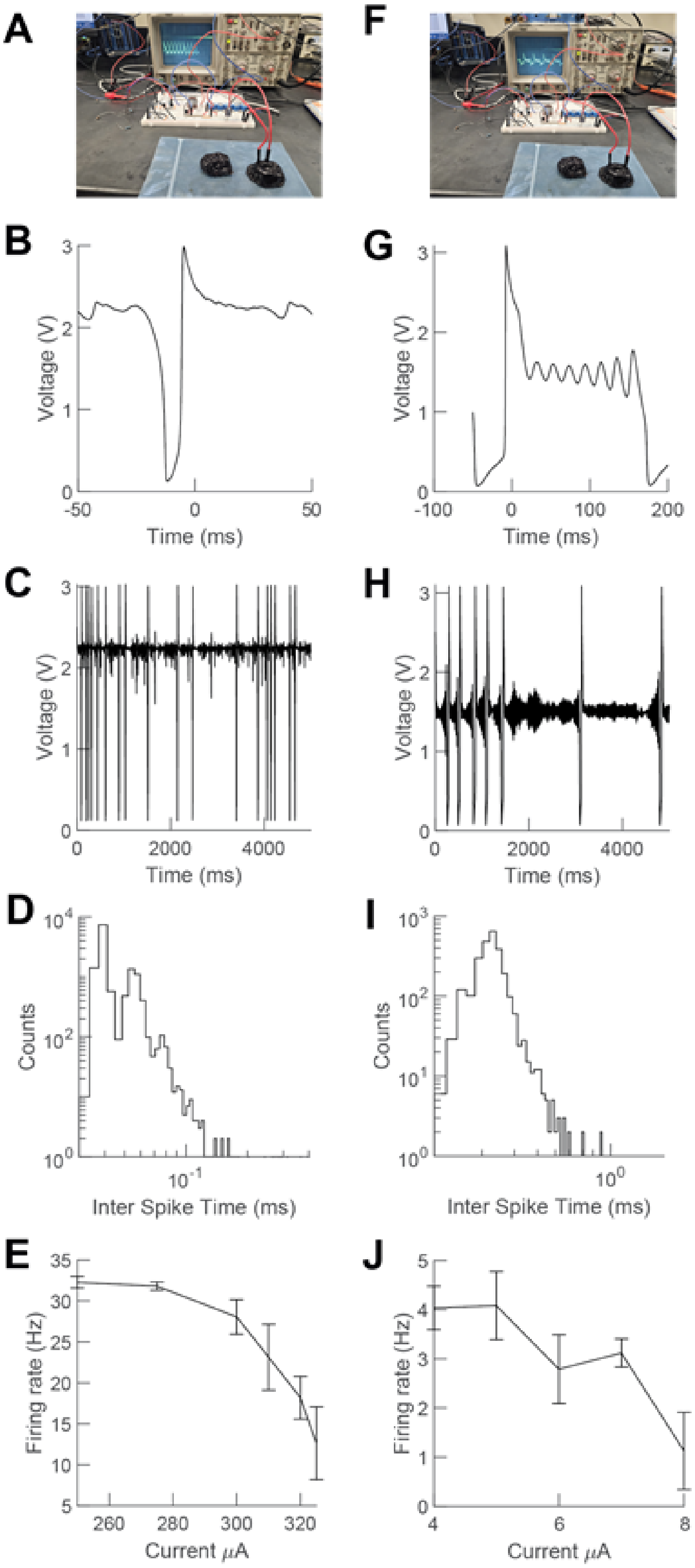

Figure S6. The fractional order Hodgkin-Huxley circuit with a dried fruit. A) The circuit generated spikes when connected to a dried pitted prune. B) The circuit generated action potentials. C) The spike trains showed random activity. D) The inter-spike-interval. E) The average firing rate as a function of input current. F-J) same measurements as in A-E when the electrodes were inserted farther apart on the prune.

1. Tsirimokou G, Psychalinos C, Elwakil AS, Salama KN. Electronically tunable fully integrated fractional-order resonator. IEEE Transactions on Circuits and Systems II: Express Briefs. 2017;65(2):166-70.
2. Elwakil AS. Fractional-order circuits and systems: An emerging interdisciplinary research area. IEEE Circuits and Systems Magazine. 2010;10(4):40-50.
3. AboBakr A, Said LA, Madian AH, Elwakil AS, Radwan AG. Experimental comparison of integer/fractional-order electrical models of plant. AEU-International Journal of Electronics and Communications. 2017;80:1-9.
4. Jesus IS, Tenreiro Machado J, Boaventure Cunha J. Fractional electrical impedances in botanical elements. Journal of Vibration and Control. 2008;14(9-10):1389-402.
5. Roy A, Mallick A, Das S, Aich A. An experimental method of bioimpedance measurement and analysis for discriminating tissues of fruit or vegetable. AIMS Biophysics. 2020;7(1).
6. Gadallah SI, Ghoneim MS, Elwakil AS, Said LA, Madian AH, Radwan AG. Plant Tissue Modelling Using Power-Law Filters. Sensors. 2022;22(15):5659.
7. Mohsen M, Said LA, Madian AH, Radwan AG, Elwakil AS. Fractional-order bio-impedance modeling for interdisciplinary applications: A review. IEEE Access. 2021;9:33158-68.
